# Unveiling the Role of Inflammation and Oxidative Stress on Age-Related Cardiovascular Diseases

**DOI:** 10.1155/2020/1954398

**Published:** 2020-05-08

**Authors:** Arthur José Pontes Oliveira de Almeida, Mathania Silva de Almeida Rezende, Sabine Helena Dantas, Sonaly de Lima Silva, Júlio César Pinheiro Lúcio de Oliveira, Fátima de Lourdes Assunção Araújo de Azevedo, Rayanne Maira Felix Ribeiro Alves, Geovânia Maria Sales de Menezes, Pablo Ferreira dos Santos, Tays Amanda Felisberto Gonçalves, Valérie B. Schini-Kerth, Isac Almeida de Medeiros

**Affiliations:** ^1^Departamento de Ciências Farmacêuticas/Centro de Ciências da Saúde, Universidade Federal da Paraíba, Cidade Universitária-Campus I, Caixa Postal 5009, 58.051-970 João Pessoa, PB, Brazil; ^2^INSERM (French National Institute of Health and Medical Research), UMR 1260, Regenerative Nanomedicine, Faculty of Pharmacy, University of Strasbourg, 67000 Strasbourg, France

## Abstract

The global population above 60 years has been growing exponentially in the last decades, which is accompanied by an increase in the prevalence of age-related chronic diseases, highlighting cardiovascular diseases (CVDs), such as hypertension, atherosclerosis, and heart failure. Aging is the main risk factor for these diseases. Such susceptibility to disease is explained, at least in part, by the increase of oxidative stress, in which it damages cellular components such as proteins, DNA, and lipids. In addition, the chronic inflammatory process in aging “inflammaging” also contributes to cell damage, creating a stressful environment which drives to the development of CVDs. Taken together, it is possible to identify the molecular connection between oxidative stress and the inflammatory process, especially by the crosstalk between the transcription factors Nrf-2 and NF-*κ*B which are mediated by redox signalling and are involved in aging. Therapies that control this process are key targets in the prevention/combat of age-related CVDs. In this review, we show the basics of inflammation and oxidative stress, including the crosstalk between them, and the implications on age-related CVDs.

## 1. Introduction

The average life expectancy of the global population has been increasing rapidly [1]. According to the United Nations (UN), the world population over 60 years will keep growing exponentially in the next decades, rising from 12%, data from 2015, to 22% in 2050 [2]. In parallel, age-related diseases have also been increasing, highlighting cardiovascular diseases (CVDs) as the main cause of morbidity and mortality worldwide, aging being the main risk factor for these diseases [3–5].

**Figure 1:**
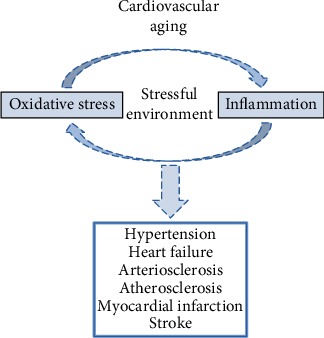
Aging and CVDs. The cardiac and arterial aging is characterized by a stressful environment to the cells, derived, at least in part, from the high levels of oxidative stress and chronic inflammation. The crosstalk between them is a vicious and gradual cycle, which accompanies the course of aging. These changes drive to the development of CVDs, such as hypertension, heart failure, arteriosclerosis, atherosclerosis, myocardial infarction, and stroke.

Cardiovascular aging (cardiac and vascular aging) is characterized by a progressive decline in physiological processes at the molecular, cellular, and tissue levels [6–8]. Two factors play a key role in this process: the gradual and persistent increase in inflammation “inflammaging” and the oxidative stress, including the crosstalk between them [9, 10]. These processes are associated with the pathophysiology of aging-related CVDs, such as hypertension, acute myocardial infarction, and stroke (Figure 1) [11–13].

This review brings an integrated approach to the role of inflammation and oxidative stress associated with the pathophysiology of age-related CVDs, addressing molecular, cellular, and physiological aspects, with the potential source of antioxidants as pharmacological tools.

## 2. Cardiovascular Aging

Cardiovascular aging is a dynamic process caused by several mechanisms that include a progressive change in the function and structure, resulting in the impairment of cardiovascular homeostasis [14]. Briefly, the vessels and the heart become more rigid and more fibrotic as we age, a factor that predisposes the emergence of CVDs [15].

### 2.1. Cardiac Aging

Cardiac aging is characterized by cardiomyocyte hypertrophy, inflammation, and the gradual development of cardiac fibrosis [16]. In addition, there is decreased elasticity and increased stiffness [17]. These changes are consequences of molecular responses to cellular stressors, such as oxidative stress and inflammation, and involve mechanisms such as ventricular wall thickness, myocardial fibrosis, and fibrocalcification, which ultimately decrease cardiac output and ventricular compliance [14, 17].

Structurally, there is an increase in deposition of collagen fibers by crosslinking, which compromises function and promote fibrosis, plus increase myocardial stiffness [14]. This increased stiffness produces an increase in the incidence of cardiac hypertrophy, developed by the participation of key mediators such as ROS/RNS and inflammation [18].

**Figure 2:**
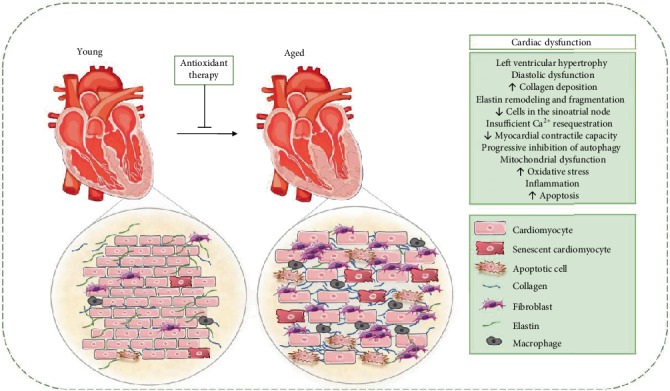
Cardiac structural and functional alterations during aging. In cardiac aging, there is a significant increase in myocardial thickness, characterized by an increase in heart size, whereas there is a decrease in the total number of cardiomyocytes. These changes alter the overall shape of the heart from elliptical to spheroid and generate greater stress on the heart wall and compromise the contractile efficiency of the heart. Fibrosis, one of the main determinants of cardiac remodelling, is characterized by increased collagen deposition, resulting in increased myocardial stiffness and, consequently, cardiac dysfunction. In the aged heart, there is diastolic dysfunction due to oxidative damage to the SERCA of the sarcoplasmic reticulum, decreasing its subsequent Ca^2+^ activity, prolonging diastolic relaxation. Finally, mitochondrial dysfunction, augmentation of inflammation and oxidative stress, and apoptotic and necrotic myocyte cell death are important determinants of the aging process, possibly mediating the occurrence of cardiac dysfunction in aging.

Left ventricular hypertrophy, evidenced by thickening of the left ventricular wall, is a compensatory reaction to the cumulative loss of myocytes. However, in order to minimize the damage from this loss, hypertrophy minimizes myocardial wall stress and may help maintain cardiac function (Figure 2) [19].

The renin angiotensin system seems to actively participate in the cardiac aging process [20]. This system mediated stimulation of ROS production via NADPH oxidase, which contributes in the stretching of myocytes and cardiac fibroblasts [21]. This triggers growth factor signalling (e.g., ANG-II/TGF-*β*), which has been shown in vitro to promote cell growth, matrix production, and increased apoptosis [22].

In addition, aging is accompanied by a decrease in the number and function of sinoatrial node pacemaker cells, with the concomitant increase in conduction abnormalities, resulting in a decrease in heart rate variability [12]. In addition, there is a variable degree of calcification and fibrosis on the left side of the cardiac skeleton. This can impact the atrioventricular node resulting in the blockade of the conduction [22].

### 2.2. Vascular Aging

**Figure 3:**
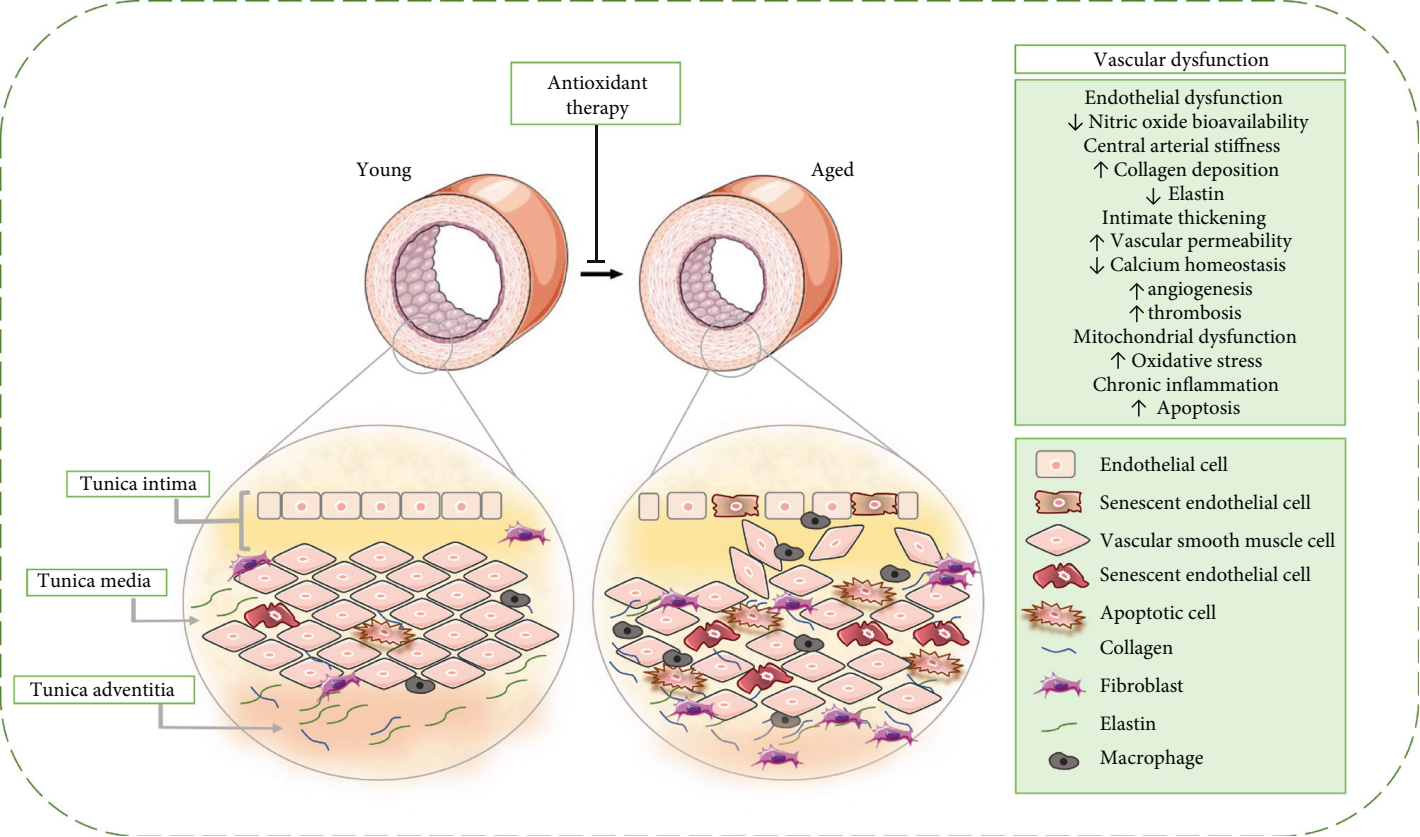
Vascular structural and functional alterations during aging. Vascular aging is associated with critical modifications of the vascular wall such as endothelial dysfunction and increased arterial thickness and stiffness. Endothelial dysfunction includes reduced vasodilatory and antithrombotic properties, with increased oxidative stress and inflammatory cytokines, increasing the risk of atherosclerosis and thrombosis. Furthermore, the endothelial barrier becomes porous and vascular smooth muscle cells migrate to subendothelial spaces and deposit extracellular matrix proteins resulting in the thickening of the tunica intima. Central arterial stiffness is related to the loss of elastic fibers and the increase of collagen accumulation in the vessel wall, which deteriorates vascular functionality. Endothelial dysfunction and arterial stiffness are mediators connected closely in vascular dysfunction during aging. If the artery is more rigid, greater will be the exposure of the endothelium to hemodynamic load, promoting endothelial activation, inflammation, and oxidative damage. Antioxidant therapies have been shown to attenuate aging-induced changes through endothelial dysfunction and changes in the extracellular arterial matrix that cause central arterial stiffness.

Arterial aging is associated with failures in signalling pathways which are essential for the physiological functioning of the vascular system [23, 24]. These failures result from dynamic changes in the mechanical, structural, and functional properties of the vascular wall, promoting the development of endothelial dysfunction, inflammation, vascular remodelling, and arterial stiffness (Figure 3) [25, 26].

The arterial wall is a heterogeneous structure composed of three layers: an intima (consists essentially of endothelial cells), middle (smooth muscle cells and elastic fibers), and adventitial tunic [27]. Each layer exhibits specific histological, biochemical, and functional characteristics, and each contributes uniquely to maintain vascular integrity [28]. Under physiological conditions, the vascular endothelium synthesizes and releases substances to modulate the arterial structure and the vasodilatory, thrombolytic, and vasoprotective functions [29]. Nitric oxide (NO) is the major mediator of normal endothelial function, mainly due to its powerful vasodilator action [30].

However, in aging, the endothelium modulatory role is not totally preserved. The mechanisms associated with age-related endothelial dysfunction are basically related to decreased bioavailability of NO [31]. The lower bioavailability of NO results from decreased synthesis or increased NO degradation, which produces vasoconstriction, inflammation, and prothrombotic effects [32]. In addition, mediators such as endothelin-1 (ET-1), angiotensin II (ANG-II), and cyclooxygenase-derived (COX) eicosanoids also compromise endothelium-dependent vasodilation, resulting in increased vasoconstrictor tone, which eventually drives to CVDs, such as hypertension [33, 34].

As the vessels age, the balance between proteases and their inhibitors is impaired, and the release of proteases, such as matrix metalloproteinase (MMPs), increases [35]. Collagen and elastin are regulated by MMPs, which, in turn, are regulated by inflammatory mediators [25]. This chronic increase in the activation of MMP (-2/-7/-9/-14) in the vessels plays a central role in the vascular remodelling associated with age [36]. In addition, increased TGF-*β* activity contributes to increased vascular rigidity through the stimulation of interstitial collagen synthesis. Such structural and functional changes directly affect cardiac homeostasis and are intrinsically related to age-related CVDs [35].

## 3. Inflammaging: Implications on the Cardiovascular System

Acute and transient inflammation is vital to life by stimulating a beneficial immune response to harmful conditions such as tissue injury or pathogen invasion [37]. This process also allows cell elimination and renewal in several tissues, including the cardiovascular system [38, 39]. However, low levels and persistent inflammation lead to tissue deterioration, which is related to the pathophysiology of CVDs, such as hypertension and arteriosclerosis [13, 40]. In addition, this stage of chronic inflammation—characterized by the increase of proinflammatory cytokines in the circulation—is one of the hallmarks of aging “inflammaging” [6, 9], being considered an important risk factor for morbidity and mortality in the elderly population [41]. On the other hand, centenarians have high levels of systemic inflammation, however, without suffering from the deleterious effects of inflammation [42]. Scientists believe that suppression of inflammation is the most important drive of successful aging [43, 44].

### 3.1. Source of Inflammaging

There are several sources of immunological stimuli which contribute to the stage of chronic inflammation characteristic of aging [9, 37]. First, the accumulation of cell debris (e.g., proteins or organelles damaged) due to failure to clean up system in aging (e.g., deficiencies in the autophagy pathways) leads to a persistent alert status by which drives the activation of the innate immune system [37, 45]. A crucial part of the innate response is the assembly of the inflammasome a cytosolic complex of proteins that activates caspase-1 [46]. Among these inflammasomes, Nlrp3 has been under intensive investigation and has been considered a crucial driver of sterile inflammation during aging [47]. Nrlp3 mediates the maturation of IL-1*β* and IL-18, both proinflammatory cytokines, that are induced by several damage-associated molecular patterns (DAMPs) and pathogen-associated molecular patterns (PAMPs) [46, 48]. An example of such a mechanism occurs when the mitochondria is damaged due to an increase in mtROS levels and mtDNA damage, which drives to Nlrp3 activation, due to the release of mitochondria-derived DAMPS [37, 49]. In addition, decreased NAD^+^ levels due to mitochondrial dysfunction are also implied in the Nlrp3 activation [50]. Moreover, autophagy and Nlrp3 activation are reversely correlated [51], which may explain the accumulation of mitochondrial dysfunction in aging [47]. On the other hand, Marín-Aguilar and colleagues showed that Nlrp3 suppression delays cardiac aging and improves longevity in mice [52].

The stage of cell senescence secretes a variety of proinflammatory cytokines, interleukins, and growth factors, known as SASP (secretory-associated senescence phenotype), being another source of inflammaging [53]. This phenomenon is explained by the senescent cells' attempt to attract cells of the immune system in order to be phagocytosed, promoting tissue regeneration [54]. However, in order for immunosenescence, the stimulus generated by the senescent cells is not able to recruit enough functional cells of the immune system, which decreases tissue homeostasis [55, 56], a long-term process that plays a negative effect on aging and age-related diseases [57]. In addition, the endothelial progenitor cells (EPC) suffer with the stem cell exhaustion process, which result in low regenerative capacity of these cells [58, 59]. The accumulation of senescent EC in aging and the dysfunction of progenitor lineages converge to vascular impairment in old age [60–62]. Recent studies have shown that senescent cell clearance from the body is a promising therapeutic target to delay aging and combat age-related CVDs [63, 64], which leads to increased health span [65].

Another source of chronic inflammation is the microbiota [66, 67]. The elderly present a decrease in microbiota diversity (decrease of commensal bacteria and increase of opportunistic bacteria) [68], which has been associated with frailty in the elderly [69]. The integrity of the gut barrier along with microbiota is the first defence against pathogens, and its deregulation may increase the infiltration to infectious agents [70, 71]. High levels of proinflammatory cytokines (e.g., TNF-*α*, IL-6, and IL-8) have been related to the changes in the microbiota [67], which is associated to a chronic systemic inflammation [72].

In addition, another source of chronic inflammation is based on an increased activation of the coagulation system in the elderly [73]. The coagulation system and inflammation have many shared pathways [74]. IL-1, IL-6, and TNF-*α* directly influence coagulation pathways, creating a reciprocal activation. They modify endothelial function, leading to a prothrombotic state with inhibition of fibrinolysis, increased production of platelet activation factors, and activation of the intrinsic and extrinsic coagulation pathways [75, 76]. Humans with exceptional longevity present also a hypercoagulation state in addition to the chronic inflammation [77]. The high incidence of thrombosis in the elderly population may be related to this characteristic phenomenon of aging [78].

**Figure 4:**
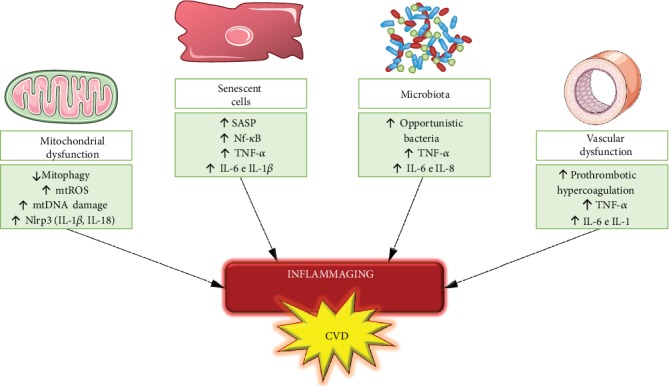
Different sources contribute to inflammation in aging. Aging is accompanied by mitochondrial dysfunction, increase in senescent cells numbers, dysregulated microbiota, and a hypercoagulation state that mediate the inflammatory processes that are characteristics of aging. Together, these processes play a key role on the development of age-related cardiovascular diseases.

Together, these factors generate continuous stimuli of the immune system, creating an unresolved inflammation stage, which is related to the pathophysiology of CVDs associated with aging (Figure 4) [79, 80].

### 3.2. Pro- and Anti-inflammatory Markers in Aging

The elderly have high levels of proinflammatory cytokines in the circulation, including IL-1*β*, IL-1 antagonist receptor (IL-1RN), IL-6, IL-8, TNF-*α*, IL-13, IL-18, IFN-*γ*, and C-reactive protein (CRP) [76]. Such cytokines mediate inflammation through the interaction with toll-like receptors (TLRs), IL-1R receptor, IL-6 receptor (IL-6R), and TNF receptor (TNFR) [81]. Activation of the receptors triggers intracellular signalling pathways, including mitogen-activated protein kinase (MAPK), nuclear kappa-B (NF-*κ*B), Janus kinase (JAK), and transcriptional activator (STAT) pathways [82, 83]. Such signalling pathways have their expressions regulated by interleukins and inflammation products, generating a fast and efficient feeding loop. In addition, high levels of these markers are correlated with senescence, endothelial dysfunction, and cardiovascular aging [84, 85].

Anti-inflammatory cytokines and soluble protein antagonist receptors act to balance proinflammatory agents in the search for homeostasis. IL-10, IL-37, and transforming growth factor (TGF-*β*) are the main examples of these cytokines [76]. IL-10 acts by suppressing levels of IL-6, IL-8, and TNF-*α*. In addition, it has been reported as an endothelial protector [86]. However, it still has controversies about its actual role in vascular homeostasis. TGF-*β* acts on acute phase responses and is involved in postinjury repair to damage or infection [87]. IL-37 limits the action of innate inflammation, decreasing the production of proinflammatory cytokines, such as IL-1*β* and TNF-*α* [88].

### 3.3. Inflammaging: Molecular Pathways Implicated on the Cardiovascular System

The transcription factor NF-*κ*B plays an important role in the control of several cellular processes such as immune response (innate and adaptive), inflammation, cell survival, proliferation, and apoptosis [89]. Although NF-*κ*B is critical for physiological homeostasis, this transcription factor is superexpressed in aging, leading to a stage of chronic inflammation “inflammaging” [45], its overexpression being related to aging-related CVDs, such as hypertension and arteriosclerosis [90, 91].

NF-*κ*B promotes the expression of various proinflammatory genes, including those coding of cytokines, chemokines, and adhesion molecules [92]. Cytokine production is often induced by molecular patterns associated with PAMPs and DAMPs, by which it acts through pattern recognition receptors (PRRs), such as TLRs and NOD-like receptors (NJRs) [93, 94]. DAMPS are released from the extracellular or intracellular spaces following cell death or tissue injury [95]. The most widely studied DAMPs are HMGB1, heat shock proteins (HSPs), and purine metabolites such as ATP and uric acid [96]. These DAMPs are recognized by macrophages, and inflammatory responses are triggered by different pathways, including TLRs and inflammasomes. Such a process is also involved in the pathogenesis of atherosclerosis due to macrophage recruitment as a result to arterial injury, which is rich in DAMPS [97].

Under physiological conditions, the activation of NF-*κ*B in response to proinflammatory signals is short lived, and the reaction stops rapidly when the signal is finalized [92]. Briefly, the transcription factor NF-*κ*B is bounded to its cytoplasmic inhibitor I*κ*B*α*. PRRs use similar signal transduction mechanisms to activate I*κ*B kinase (IKK), which is composed of two kinase subunits, IKK*α* and IKK*β*, and a regulatory kinase subunit, IKK*γ* [98]. The IKK complex can be activated by several stimuli, such as growth or stress factors, which leads to the phosphorylation of I*κ*B*α*, promoting its dissociation with NF-*κ*B [99]. I*κ*B is degraded via proteasome, while NF-*κ*B is translocated to the nucleus, binding to its *κ*B site for encoding specific genes [92].

**Figure 5:**
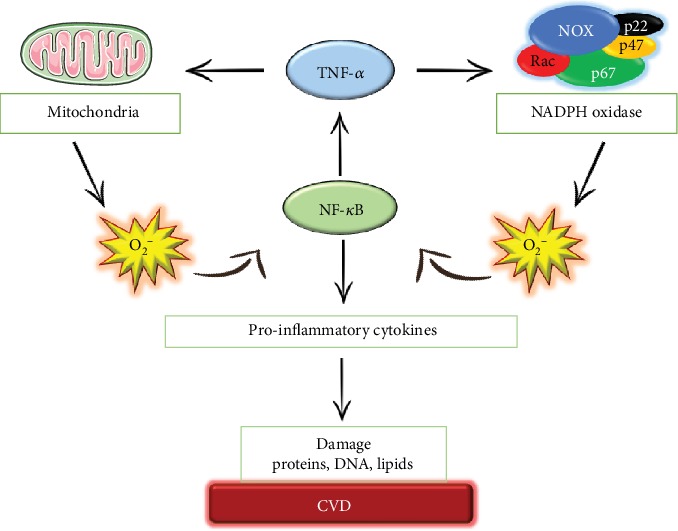
Many ROS/inflammation interactions are controlled by autoregulatory mechanisms. ROS produced by both NADPH oxidase and mitochondria induce proinflammatory release via NF-*κ*B, such as TNF-*α*, which modulates an increase in NADPH oxidase and contributes to mitochondrial dysfunction, resulting in more ROS. The upregulation of these mechanisms contributes to cellular damage and eventually drives to cardiovascular diseases.

Interestingly, many of the products encoded by NF-*κ*B are activators of this transcription factor, generating a positive feedback, which means, the more inflammatory mediators produced, the greater is the expression of NF-*κ*B [100]. This cycle is accompanied by an increase in ROS/RNS levels, generating a stress environment related to the development of age-related CVDs (Figure 5) [101, 102].

## 4. Reactive Oxygen/Nitrogen Species (ROS/RNS)

ROS/RNS are produced by all living organisms as a result of cellular metabolism [103]. Despite the organisms presenting their own antioxidant defences (e.g., enzymes, proteins, and vitamins) [104], ROS/RNS can accumulate with age due to its overproduction or failure in the antioxidant system, leading to the stage of “oxidative stress” [105]. Denham Harman, in 1956, proposed the “free radical theory of aging”, which argued that ROS derived from metabolism was the major cause of aging [106]. Since then, several publications have reported the deleterious effects of ROS/RNS on aging, as well as its relationship in the pathophysiology of CVDs [107–110].

### 4.1. Source of ROS/RNS

There are several sources of ROS/RNS present on the cardiovascular system, such as mitochondria and NADPH oxidases (NOX), the major sources of intracellular ROS/RNS [111]. Interestingly, both sources have interactions between them, which contribute to the gradual progression of oxidative stress [112, 113]. NOX also has its regulation increased by TNF-*α* [114], as well as activation of the AT1 receptor by the agonist angiotensin II [115], connecting, at least in part, oxidative stress with the inflammatory process and vascular dysfunction [116]. Other sources of ROS/RNS are uncoupled NO synthase, cytochrome p450, xanthine oxidase, the endoplasmic reticulum, peroxidases, cyclooxygenases, lipid oxidases, and some hemoproteins [117].

### 4.2. Examples of ROS/RNS

A range of molecules with oxidizing properties contributes to the regulation of cellular redox potential [118]. These ROS/RNS include superoxide anions (O_2_^·−^), radical hydroxyl (HO^·^), nitric oxide (NO^·^), and lipid radicals, which present unpaired electrons, being considered free radicals [119]. Other ROS/RNS such as hydrogen peroxide (H_2_O_2_), peroxynitrite (ONOO¯), and hypochlorous acid (HOCl) are not free radicals but have oxidizing effects that contribute to oxidative stress [120].

O_2_^·−^ and H_2_O_2_ are produced enzymatically and are involved in physiological signalling as well as the pathologies associated with oxidative stress [121]. In addition, O_2_^·−^ can be converted to H_2_O_2_ (less toxic) by superoxide dismutase (SOD) [122]. H_2_O_2_ acts as a second messenger, regulating redox signalling at homeostatic physiological levels [123–125]. Their levels ensure an adaptive response to stress, which is necessary for cellular survival [126, 127].

NO^·^ is a potent vasodilator produced in the endothelium by eNOS activity [128, 129]. In addition, NO^·^ has antiplatelet, antithrombotic, and anti-inflammatory functions [28]. Endothelial health is dependent of NO^·^ levels [129, 130]. HO^·^ and ONOO¯ are not considered signalling molecules due to their highly reactive nature, but they contribute significantly to oxidative stress and tissue damage [131]. While (HO^·^) is produced by the catalysis of H_2_O_2_, ONOO¯ is produced by the reaction between (O_2_^·¯^) and (NO^·^) [104]. High levels of ONOO¯ and depletion of NO^·^ levels are related to endothelial dysfunction, an event present in the genesis of several CVDs [132].

### 4.3. Antioxidant System

In a homeostatic live system, ROS/RNS concentrations are controlled and preserved through enzymatic and nonenzymatic complexes of cellular detoxification, known as antioxidants [133, 134].

Nrf-2 (nuclear factor 2 related to erythroid factor 2), a transcription factor sensitive to redox potential, is the main regulator of the antioxidant system present on the cardiovascular system [135]. Under physiological conditions, Nrf-2 is downregulated in the cytoplasm by the Keap1 (Kelch-like ECH-associated protein-1) protein, leading to ubiquitination and consequent degradation via the proteasome [136–138]. Under stress conditions, Nrf-2 dissociates from the complex and translocates to the nucleus, binding to the AREs (antioxidant response elements), encoding the transcription of antioxidant enzymes and phase II detox, such as superoxide dismutase (SOD), catalase (CAT), glutathione peroxidase (GPx), glutathione reductase (GR), heme oxygenase-1 (HO-1), and NAD (P) H quinone oxidoreductase-1 (NQO1) [139, 140]. The cytoprotective effect of Nrf-2 is related to its antioxidant and anti-inflammatory properties [141, 142].

FOXO (Forkhead box O) is another family of transcription factors that present cytoprotective effects [143]. FOXO3, a member of the FOXO family, has been associated with the regulation of oxidative stress, attenuating ROS/RNS through the transcriptional activation of SOD and CAT [144]. Together, Nrf-2 and FOXO3 are the major transcription factors involved in cell protection [145, 146]. Thereby, they are very promising pharmacological targets for age-related CVDs [147–149].

## 5. Crosstalk between Nrf-2 and NF-*κ*B

**Figure 6:**
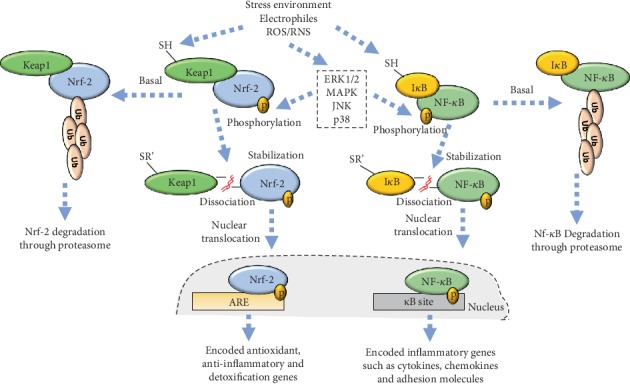
The crosstalk between Nrf-2 and NF-*κ*B transcription factors. The stress environment caused by oxidative stress leads to functional modifications in both Nrf-2 and NF-*κ*B transcription factors. Oxidation occurs at the thiol's sites of cysteine residues, allowing dissociation with their cytosolic inhibitors (Keap1 and I*κ*B, respectively), leading to nucleus translocation and consequent encoding of target genes. Oxidative stress can interfere in both pathways indirectly by activating signalling pathways, such as ERK1/2, MAPK, JNK, and p38, which leads to the phosphorylation of these transcription factors. However, in basal situations, Nrf-2 and NF-*κ*B are degraded via proteasome, which maintain homeostasis.

The change in the expression of Nrf-2/Keap1 and NF-*κ*B is gradual and follow the course of cardiovascular aging [7]. In addition to acting individually on the redox signalling cascade, both interact with each other, guiding the communication between oxidative stress and inflammation (Figure 6) [123, 150].

Transcription factors such as Nrf-2 and NF-*κ*B are not directly oxidized but are translocated to the nucleus due to the oxidation of their cytosolic inhibitors (Keap1 and I*κ*B, respectively), which are redox sensitive at their cysteine sites [151]. Keap1, which has its protein structure modified by the oxidation in the cysteine residues, loses the affinity for Nrf-2, allowing its accumulation in the cytoplasm and favouring its translocation to the nucleus [136, 137, 152].

HO-1, an antioxidant enzyme encoded by Nrf-2, mediates the inhibition of NF-*κ*B [153]. Elevated levels of HO-1 in endothelial cells lead to decreased adhesion molecules mediated by NF-*κ*B, such as E-selectin and VCAM-1 (vascular cell adhesion molecule 1) [150]. In addition, metabolites derived from HO-1 inhibit NF-*κ*B translocation [154]. In contrast, excessive NF-*κ*B expression showed lower HO-1 levels, revealing NF-*κ*B suppressive activity, at least in part, on Nrf-2 transcription [155].

An oxidizing environment promotes the oxidation and degradation of I*κ*B, favouring the activation of NF-*κ*B [156]. On the other hand, a reducing environment has the opposite effect. This reducing environment is favoured by the action of GSH, an enzyme dependent on the Nrf-2 action. In fact, mice deficient in the Nrf-2 gene have a high susceptibility to high production of NF-*κ*B-dependent proinflammatory agents, such as TNF-*α*, iNOS, and COX-2 [157].

Evidence shows the suppression of the transcriptional activity of Nrf-2 by NF-*κ*B. The N-terminal region of the p65 (Rel-A) subunit of NF-*κ*B binds physically to Keap1, promoting its translocation to the nucleus and consequent inhibition of the Nrf-2/ARE pathway [158]. In addition, NF-*κ*B competes for CBP (CREB binding protein), a coactivator common to Nrf-2 near the ARE promoter region [159]. Thimmulappa and colleagues showed that knockdown mice for Nrf-2, in response to LPS, significantly increased the transcriptional activity of NF-*κ*B [160]. Taken together, these studies suggest a tight connection between ROS/RNS and inflammation signalling, due in large part, to the many interactions evolving Nrf-2 and NF-*κ*B pathways.

## 6. Age-Related Cardiovascular Diseases

Chronological age is the major risk factor for the development of CVDs [161]. In fact, this is due not only to the increase in conventional risk factors in the elderly but also to the independent and inevitable effect of aging itself [162]. The elderly, for example, are more susceptible to oxidative stress due to reduced efficiency of their endogenous antioxidant systems, favouring the activation of inflammatory pathways, which contributes to the gradual and chronic inflammation that accompanies aging [7]. These and other aging-induced changes reduce the functionality of the cardiovascular system by preventing stress response and cardioprotective interventions, making the individual more vulnerable to the development of CVDs [18].

### 6.1. Hypertension

Hypertension is characterized by persistent increase in blood pressure (BP), being one of the most common morbidities among the elderly [163]. It is a fact that this clinical condition increases due to age, which can be explained by the physiological and morphological changes in the cardiovascular system that occur during the aging process [164]. This clinical condition affects 1.13 million people worldwide and is considered one of the leading causes of death associated with chronic noncommunicable diseases [165] and a major risk factor for the development of cardiovascular complications such as stroke, AMI, heart failure, aortic aneurysm, and renal failure in this population [166]. A study by Mozaffarian and colleagues demonstrated that a high percentage of older people (70%) have hypertension compared with younger adults (32%) between 45 and 54 years [167].

The effect of aging on the vascular system is mainly marked by an increase in arterial thickness and stiffness, leading to a decrease in vascular tone [168]. These vascular changes contribute to increased procontractile response in vascular smooth muscle cells (CMLV), leading to increased BP [169].

Chronic inflammation and oxidative stress are common points between the biology of aging and CVDs [170]. Endothelial dysfunction—triggered by both processes—leads to a higher production of contracting factors (TXA2, PGH2, and ET-1) in detriment to the relaxing factors (NO, PGI2, and EDHF), contributing to a persistent vasoconstriction and consequent elevation of BP [171, 172]. This process leads to arterial stiffening due to collagen accumulation and a decrease in the amount of elastin, which decreases the ability of blood vessel to respond to stimuli [32].

Chaudhary and his group demonstrated that hypertensive patients over 60 years of age had a higher oxidative stress index and lower total blood antioxidant and mean arterial flow dilation rates than hypertensive patients under 60 years of age [173]. In addition, in EC, there is a change in proinflammatory phenotype triggering an increase in the expression of inflammatory cytokines, adhesion molecules, and chemokines that activate NF-*κ*B signalling [60]. An increase in these factors leads to the infiltration of T cells and macrophages, which contributes to tissue damage [174]. In addition, increased cytokines such as interferon-*γ*, IL-1*β*, and TNF-*α* lead to increased oxidative stress in CMLV and EC [175]. Thus, it is noted that oxidative stress and the inflammatory process participate as essential components to affect the function of the vascular system, initiating a vicious cycle between increased BP, vascular remodelling, and stiffness, characterizing the state of continuous hypertension on the aging process [176].

### 6.2. Heart Failure

Heart failure (HF) is defined as a chronic and progressive clinical condition in which the heart cannot pump blood to meet the necessary tissue metabolic needs [177]. HF has an increased prevalence with age, with incidence rates <1% in individuals under 50 years, reaching 30% in individuals with advanced age (>80 years), being considered a disease of the elderly [178].

Morphological and functional changes in the heart due to aging explain the high rates of HF in old age [179]. The mechanisms involved behind these changes include (1) high levels of oxidative stress and inflammation, (2) high rate of apoptosis, (3) loss of regenerative capacity of cardiac progenitor cells, (4) hypertrophy of remaining cardiomyocytes, (5) loss in mitochondrial health, and (6) unbalance of calcium homeostasis. Such events are highly connected with HF [180–182].

Structural changes were observed regarding the reduction in the number of cardiomyocytes associated with their hypertrophy, collagen accumulation, and its metabolites, and functionally, it is possible to verify change in the maximum ejection fraction [183]. These hallmarks contribute to the aggravation of HF in elderly patients.

### 6.3. Arteriosclerosis-Atherosclerosis

Arteriosclerosis is defined by the formation and growth of plaques in the arterial lumen with consequent loss of vascular elasticity, leading to reduced blood flow in the affected vessel [184]. This clinical condition increases dramatically in the elderly, even in the absence of classical risk factors such as dyslipidemia [185]. Recently, atherosclerosis has come to be considered an inflammatory disease [186–188], affecting mainly the wall of large and medium arteries [189]. The formation and progression of atherosclerotic plaques support the “injury response” hypothesis of this disease, implying that plaque formation is a consequence of the local endothelial lesion associated with the inflammatory process [190, 191]. Metabolic syndrome, abdominal obesity, dyslipidemia, insulin resistance, hypertension, serum total and LDL cholesterol, smoking, and aging are related to the development of atherosclerotic lesions in arterial wall [184, 192].

The process to formation of atherosclerosis involves damage in vascular endothelial cells through oxidative stress, cytokines, and chemokines [193, 194]. These chemokines attract monocytes from blood circulation to the injured area which attach to the endothelium through interaction with adhesion molecules. Posteriorly, the monocytes enter in the subendothelial space, where it differentiate itself in macrophages who release cytokines. The high levels of low-density lipoprotein (LDL) infiltrate the vascular intima where it is oxidized. Then, the macrophages take up the oxidized LDL and form foam cells and atherogenesis. The vascular smooth muscle cells present in the media layer transform and migrate into the intima layer; after, these cells proliferate and produce extracellular matrix, where they also contribute to atherogenesis [195]. The rupture on this plaque leads to myocardial infarction and stroke.

ROS are involved in the development of atherosclerosis in a wide aspect. ROS facilitate inflammatory cell recruitment and lipid deposition in the intimal layer through the increase of adhesion molecules as ICAM-1 and VCAM-1. ROS also are related with the increase of proliferation of vascular smooth muscle cells [196, 197]. In addition, the ROS/RNS-rich environment associated with inflammation leads to elastin depletion, collagen accumulation, and immune recruitment, leading to disease progression [198, 199].

Proinflammatory cytokines also have an important role in atherosclerosis. They contribute to the formation of macrophages in the subendothelial space. Important cytokines such as TNF-*α*, IL-1, IL-6, IL-12, and NF-*κ*B are related to atherosclerosis [193]. Kirii and colleagues evaluating the role of IL-1*β*, found, through experiments with mice, that the percentage of atherosclerotic lesions at aortic sinus was less in mice lacking IL-1*β* compared with wild mice [200]. Brånén and colleagues showed that mice lacking TNF-*α* had a reduction of 50% of the lesion compared to the control [201]. Childs and his group found in the experiments with mice that macrophages accumulate in the subendothelial space and increase the expression of inflammatory cytokines and chemokines [202]. Taken together, aging is an important factor to atherosclerosis because it accelerates the structural and compositional modifications of the vessel.

### 6.4. Myocardial Infarction

Myocardial infarction (MI) is the cardiovascular disease responsible for the highest incidence of mortality in old age [203]. MI occurs as a result of an interruption of blood supply to the heart, resulting in a lack of oxygen to the heart muscle (ischemia), which leads to the cardiomyocytes' death, triggered by coronary artery atherosclerosis [204]. Furthermore, this clinical issue highly increases with advancing of age. The mean age for the development of myocardial infarction is 65 years for men and 72 years for women [205].

Some studies show a tight connection between aging and its consequences for the genesis or aggravation of heart infarction. However, these studies are still scarce. It is established in the literature that aging is related to better heart vascularization, which prevents total organ ischemia in a patient from acute ischemic condition. On the other hand, aging leads the heart to damage, such as oxidative stress and inflammation, which have an influence on the development of ischemia and, ultimately, to MI.

The higher production of ROS in the cardiomyocytes is directly related to the sudden increase of Ca^2+^ in the cytoplasm, which directly contributes to the apoptosis and necrosis of ischemic cardiomyocytes, raising tissue damage. Waypa and colleagues demonstrated that hypoxia triggers increased intracellular Ca^2+^ concentration [206]. This effect is due to Ca^2+^ release from the sarcoplasmic reticulum and K_v_ inhibition, which triggers plasma membrane depolarization and Ca_v_ activation, allowing calcium influx to cytosol [207].

Both ROS and Ca^2+^ are capable of triggering the process of cellular apoptosis by opening the mitochondrial permeability transition pore (MPTP), which will lead to loss of mitochondrial shape, swelling, dissipation of membrane potential, and oxidative phosphorylation, in addition to the leakage of mitochondrial content [208, 209]. These factors together increase cell death in reperfusion, which, in short, increases the area of cardiac necrosis.

In animal infarction models, one of the most prominent findings is oxidative stress, which is detected by decreasing markers such as SOD and NO, plus the elevation of others such as serum MDA [210]. In addition to these classic markers, there is already evidence that other molecules are present as markers of oxidative stress in postinfarction patients, highlighting the decrease in serum thiol and disulphide levels in infarcted patients [211].

### 6.5. Stroke

Stroke is caused when there is an abrupt interruption in the blood supply to the brain that can lead to irreversible brain damage [212]. Age is the most important risk factor for developing a stroke [213] and considered the second leading cause of death worldwide [214]. With the aging of the population, as well as the increasing prevalence of underlying risk factors, there is an increased incidence of this disease, reaching about 69% of individuals over 65 years, and a prevalence of 34% in individuals over 75 years old [215].

The occurrence of acute stroke is closely related to atherosclerosis and hemodynamic changes. Blocking of the cerebral blood flow leads to hypoxia and glucose deprivation, activating different cascades of molecular signalling, which include depolarization of neurons, increased Ca^2+^ influx, ATP depletion, excitatory NT release [216], and increased expression of the hypoxia inducible factors (HIF) [217]. Increased activity of the glutamate receptor leads to an increase in intracellular Ca^2+^ levels, activation of NADPH oxidase signalling, mitochondrial dysfunction, and neuronal death [212]. The accumulation of HIF and ROS in neuronal cells generate different mechanisms involved in cell survival. HIF is related to neuroprotective effects in neuronal cells; however, in endothelial cells, it is related to the rupture of the blood brain barrier [217], whereas, ROS generate the activation of NF-*κ*B, MAPK, and the upstream pathway MMPs, in addition to increasing the expression of VEGF and its receptors [218]. Thus, the increase of ROS is involved in different mechanisms in the pathophysiology of stroke, mainly affecting nutrient transport and tissue metabolism [219]. Neuronal cell death is the main injury caused by stroke, and its mechanism involves oxidative stress, inflammation, ischemia, hypoxia, calcium ion dysregulation, and apoptosis [220, 221].

## 7. Pharmacological Interventions

Research on therapies aimed at delaying aging and preventing age-related diseases, such as CVDs, have been a great interest [222] and are mainly related to the study of pharmacological tools used in clinical therapy as well as related to substances derived from natural products [223]. Many of them interact with oxidative stress and inflammation, delaying both conditions.

### 7.1. Antioxidants as Pharmacological Tools

Antioxidants usually scavenge ROS/RNS levels minimizing the oxidative damage caused by these molecules, plus reducing inflammation by downregulating the NF-*κ*B pathway.

Exogenous antioxidants include ascorbic acid (vitamin C), which scavenges hydroxyl and superoxide radical anion, *α*-tocopherol (vitamin E), which is involved against lipid peroxidation of cell membranes, and phenolic antioxidants, which include stilbene derivatives (resveratrol, phenolic acids, and flavonoids), oil lectins, selenium, zinc, and drugs such as acetylcysteine [104].

Some vitamins have an important antioxidant function as vitamin A and its precursors *β*-carotene, vitamin C, and vitamin E [224]. Several large observational studies were conducted on the effect of intake of different vitamins and on the risk of CVDs, suggesting that higher intake of these vitamins significantly lowered the risk of these pathologic conditions [225]. Vitamin C is the major hydrophilic antioxidant and a powerful inhibitor of lipid peroxidation. In membranes, this molecule rapidly reduces 𝛼-tocopheroxyl radicals and LDL to regenerate 𝛼-tocopherol and inhibit propagation of free radicals. Vitamin E is the main hydrophobic antioxidant in cell membranes and circulating lipoproteins. Its antioxidant function is strongly supported by regeneration promoted by vitamin C. Vitamin E is thought to prevent atherosclerosis through inhibition of oxidative modification. Coenzyme Q (ubiquinol, CoQ) and lipoic acid in their reduced forms and melatonin are also efficient antioxidants [226].

In cardiovascular diseases, long-term treatment with antioxidants (vitamin C, vitamin E, coenzyme Q10, and selenium) significantly increased large and small artery elasticity in patients with multiple cardiovascular risk factors [227]. Li and colleagues showed the antioxidant benefits of individual and combined treatments of selenium, vitamin E, and purple carrot anthocyanins on D-galactose-induced oxidative damage in the blood, liver, heart, and kidney of rats [228].

Some polyphenols also play a key role in preventing CVDs. They are secondary metabolites derived from plants, characterized by the presence of an aromatic ring attached with a hydroxyl group [229]. Different studies have demonstrated the beneficial role of polyphenols, mainly related to the reduction of oxidative stress, which are implicated to the reduction of the incidence of age-related CVDs [10].

Quercetin, one of the most widely used polyphenols in the human diet, has been shown to be beneficial in preventing CVDs by acting on a wide variety of signalling pathways [230]. Studies have shown that quercetin has a protective effect on oxidative stress by reducing the activity of NADPH oxidase [231] and increase the levels of GSH, SOD, and catalase [232], in addition to reducing the expression of proinflammatory cytokines such as TNF-*α* and IL-1*β* [233].

Resveratrol also stands out as having a potent antioxidant action and has been proposed with a strong candidate in minimizing the effects associated with aging [234, 235]. Treatment with resveratrol in middle-aged rats has been shown to improve endothelial dysfunction associated with aging, probably by increasing NO and reducing ROS production [236]. In addition, resveratrol was able to reduce the production of inflammatory cytokines and chemokines through the inhibition of NF-*κ*B, contributing to protection from age-related endothelial dysfunction [237]. Resveratrol supplementation in humans, aged 30 to 70 years, over a period of 8 weeks, showed a positive upregulation of the SOD and Nrf-2 genes [238]. Additional evidence shows that resveratrol promotes longevity and cardiac performance by causing hypoacetylation of proteins that control autophagy [239].

Curcumin, a yellow polyphenol found in *Curcuma longa* rhizome, has also been reported to have antiaging properties [240]. Recently, Santos-Parker and colleagues test 12-week supplementation with curcumin in healthy elderly. At the end of treatment, it was possible to observe the improvement of endothelial function due to an increase in NO bioavailability and reduction of oxidative stress [241]. Mechanistic studies have revealed superoxide dismutase, heme-oxygenase-1, and nuclear factor erythroid 2-related factor 2 as emerging targets for the beneficial effects of curcumin on the vasculature [242]. However, although different studies have already reported their antioxidant activity, additional studies are needed to elucidate the role of this polyphenol in the process of cardiovascular aging.

The literature studies have proven the molecular mechanisms of action of grape and red wine polyphenols against oxidative and inflammatory processes [229, 243, 244]. Polyphenols inhibit the phosphorylation of MAP kinases, causing a blocking effect on the transcription factors NF-*κ*B and AP-1 and, consequently, blocking the synthesis of TNF-*α*, interleukins, chemokines, and molecule adhesion. Moreover, it can inhibit the activity of the cyclooxygenase and lipoxygenase enzymes [245]. Another proposed mechanism is the action of resveratrol on the activity of histone deacetylases, as SIRT-1 [246]. These actions together reduce the oxidation of LDL-c and the inflammatory process, attenuating CVDs [244, 247, 248].

Dietary supplementation with antioxidants has become popular. However, their biochemical mechanisms of protection against oxidative stress and antiaging effects are not fully understood. In addition, the antioxidants as pharmacological tools may be of great interest for future studies, especially in the promotion of healthy aging.

### 7.2. Metformin

Metformin is the most widely prescribed oral antidiabetic agent [249]. Mechanistically, metformin activates AMP-activated kinase (AMPK), a master regulator of metabolic homeostasis [250]. AMPK has been implicated in the control of autophagy, inflammation, mitochondrial dysfunction, and cell survival, being an important target for a healthy aging [251]. Regarding the cardiovascular system, treatment with metformin has been suggested as a potential target in the reversal of endothelial dysfunction, by inducing increased NO production mediated by MAPK through eNOS phosphorylation [252]. Moreover, metformin has been shown to improve endothelial function *in vivo* by reducing the production of superoxide anions through AMPK/PPAR*δ* activation pathway [253]. Recent studies showed that activation of AMPK through metformin has also been shown to modulate the level of oxidative stress caused by hyperglycemia, which inhibits NADPH oxidase activation [254]. Moreover, metformin positively regulates the expression of GPx by reducing ROS formation through Nrf-2 activation [255].

In addition to the regulation of the oxidative stress, metformin is involved in the control of inflammation [256]. Its contribution to the reduction of inflammation is mediated by the release inhibition of IL-6 and IL-8 in smooth muscle cells, macrophages, and endothelial cells [257]. In addition, in smooth muscle cells receiving metformin treatment, the reduction in NF-*κ*B activation and nuclear translocation was observed, as well as suppression of the proinflammatory phosphokinase AKT, p38, and ERK [257, 258].

### 7.3. Acetylsalicylic Acid (ASA)

ASA is considered one of the most promising substances for antiaging [222]. This molecule is indicated as antiplatelet therapy when used in low doses (75-100 mg daily) through the inhibition of hyperfunctional platelets, which contributes to prevent cardiovascular events such as thrombosis and atherosclerosis [259]. These effects are explained by COX-1 acetylation, leading to a reduction on thromboxane A2 production [260, 261]. In addition, ASA has an anti-inflammatory activity, through mechanisms involving COX inhibition and indirect modulation of the NF-*κ*B pathway [260]. Bode-Böger and colleagues showed that ASA prevents endothelial senescence by improving NO release and reducing ROS levels [262].

From a clinical point of view, ASA has been demonstrated to reduce CVD mortality following long-term use and is currently being recommended for patients with postacute myocardial infarction [263, 264]. On the other hand, recent studies challenge the role of ASA on the prevention of cardiovascular events in aging [265]. Thus, futures studies are necessary to clarify the benefits of ASA in old age.

### 7.4. Statins

Statins are commonly used drugs for the treatment of dyslipidemia and, in particular, hypercholesterolemia due to their properties to reduce cholesterol synthesis and subsequently the LDL cholesterol level [266]. They are also recommended for primary and secondary prevention of cardiovascular disease. Clinical studies have shown that the use of statins reduces the risk of coronary syndrome and thromboembolic disease [267, 268]. The cardiovascular beneficial effect of statins has been attributable, at least in part, to their ability to reduce oxidative stress, to regulate the eNOS/NO pathway, and to reduce the level of inflammatory markers [269, 270]. Atorvastatin reduced also glyceraldehyde-derived formation of advanced glycation end products (AGEs) in patients with AMI, providing protection to the cardiovascular system [271]. Fluvastatin increased heme-oxygenase 1 (HO-1) expression and reduced AGE-induced proliferation of VSMC, probably by the upregulation of ERK5 through the Nrf-2 pathway [272]. Statins have also been shown to be important in reducing inflammatory markers [270]. Many of the pleiotropic properties of statins have been explained by their interference with the synthesis of isoprenoid intermediates [273]. The statin-induced inhibition of prenylation of Ras protein has been linked to the reduction of the inflammatory marker C-reactive protein (CRP) [274]. These effects of statins can contribute to reduce cardiovascular diseases, especially on the elderly population.

### 7.5. Rapamycin

Rapamycin is an inhibitor of mTOR, traditionally used as an immunosuppressant; however, recent studies show its use associated with cardioprotective effects related to the aging process [275, 276]. Lesniewski and colleagues showed that 6-8-week treatment with rapamycin in elderly mice has been shown to improve endothelial dysfunction by mTOR inhibition, plus the increase of NO bioavailability and reducing NADPH oxidase expression, which ameliorated oxidative stress [277]. Evaluating the effect of rapamycin treatment on middle-aged mice, rapamycin showed reduced mitochondrial ROS production and increased the gene expression of different endogenous antioxidants, such as SOD and GSH reductase [278, 279]. It was further noted that inhibition of mTOR mediated by rapamycin protected aged endothelial cells from oxidative stress and apoptosis induced by low shear stress [280]. In addition to the effects on oxidative stress, evidence shows that treatment with rapamycin can negatively modulate the inflammatory processes by reducing TNF-*α* expression and genes involved in NF-*κ*B signalling [281]. Therefore, treatment with rapamycin has shown promise in the prevention of age-related CVDs [282].

## 8. Conclusions and Future Directions

With the increase in global life expectancy, studies on human aging have recently gained notoriety. Researchers have noted that activating longevity genes could prevent/combat many age-related diseases, increasing the health years of life “health span”. In fact, there is a bridge between the molecular mechanisms of aging and those that drive to chronic diseases, such as cardiovascular diseases. Two key points that circulate in the bridge are oxidative stress and persistent inflammation. Although its deleterious effects have been massively described, there is currently a search for understanding the reason for oxidative stress and persistent inflammation increase in aging and CVDs. In turn, persistent inflammation has several causes, which leads to damage on the cardiovascular system. An observed paradox occurs in the centenarians, which present high levels of persistent inflammation, but without suffering from its harmful effects. Such observations lead us to believe that the increase of oxidative stress and the inflammatory process has a beneficial proposal. However, it persistently leads to the disorders related with the development of CVDs in old age.

In this perspective, therapies that act in the control of oxidative stress and inflammation, however, without inhibiting their physiological functions are promising targets in the search for a healthy aging.
